# Supplementary material for: The Vector Competence of Asian Longhorned Ticks in Langat Virus Transmission
Source: Viruses. 2024 Feb 16;16(2):304. doi: 10.3390/v16020304 (PMC10893034; doi:10.3390/v16020304)
Supplement: Supplementary file 1 [file viruses-16-00304-s001.zip › viruses-2796009-supplementary.pdf]

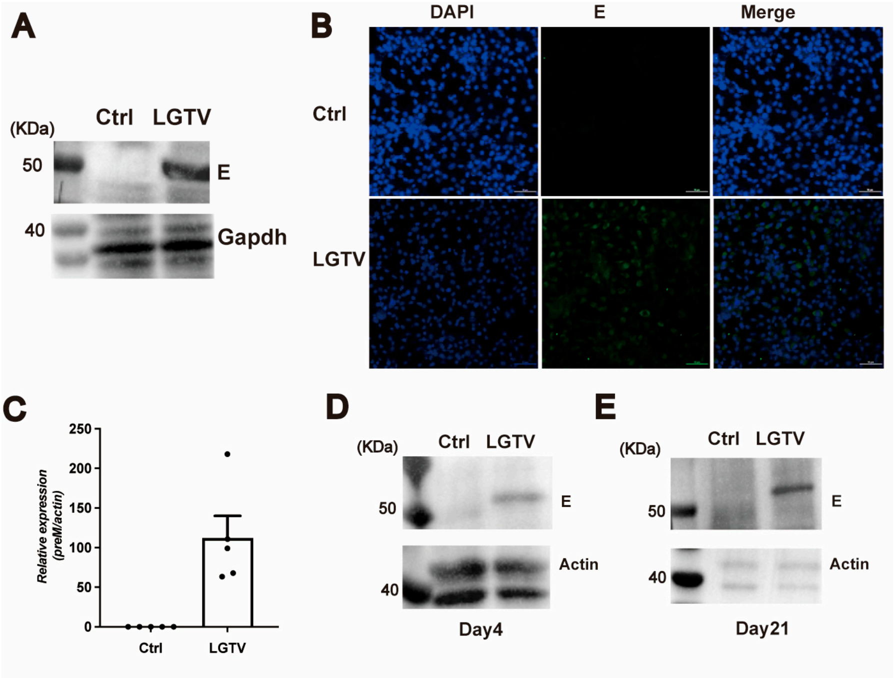

Figure S1. Validation of LGTV anti-EDⅢ.

(A)Detection of E protein in infected BHK21 cells. Lysates of infected cells were collected and analysed by western blot.

(B) Immunofluorescence analysis of E protein. BHK21 cells were infected with LGTV with moi=0.1. Immunofluorescence assay was performed on BHK21 cells 24 h post infection. Rabbit polyclonal anti-LGTV-EDIII (1:300) and Alexa Fluor 546 F(ab′)2 fragment of goat anti-rabbit IgG (1:1000) were used.

(C) LGTV detection in ticks by qPCR. Nymphs were infected with 150pfu LGTV via anal pore microinjection. Quantification of preM gene expression was performed 4 dpi by qPCR using a standard curve.

(D) and (E) LGTV detection by western blot. The infection prevalence of LGTV in ticks was 100% based on qPCR (C). To determine the specificity of the anti-E antibody, western blot was performed on ticks at 4dpi and 21dpi. Actin was used as a internal reference.
